# Persisting neuropsychiatric symptoms, Alzheimer’s disease, and cerebrospinal fluid cortisol and dehydroepiandrosterone sulfate

**DOI:** 10.1186/s13195-022-01139-9

**Published:** 2022-12-19

**Authors:** Sami Ouanes, Miriam Rabl, Christopher Clark, Clemens Kirschbaum, Julius Popp

**Affiliations:** 1 Old Age Psychiatry, Department of Psychiatry, University Hospital of Lausanne, Lausanne, Switzerland; 2 Department of Psychiatry, Hamad Medical Corporation, PO BOX 3050, Doha, Qatar; 3 Centre for Gerontopsychiatric Medicine, Department of Geriatric Psychiatry, University Hospital of Psychiatry, Minervastrasse 145, P.O. Box 341, 8032 Zurich, Switzerland; 4 Biopsychology, Technische Universität Dresden, Andreas Schubert Bau, Dresden, Germany

**Keywords:** Neuropsychiatric symptoms, Behavioral and psychological symptoms of dementia, Cerebrospinal fluid, Cortisol, Dehydroepiandrosterone sulfate, Alzheimer’s disease, Biomarkers

## Abstract

**Introduction:**

Neuropsychiatric symptoms are important treatment targets in the management of dementia and can be present at very early clinical stages of neurodegenerative diseases. Increased cortisol has been reported in Alzheimer’s disease (AD) and has been associated with faster cognitive decline. Elevated cortisol output has been observed in relation to perceived stress, depression, and anxiety. Dehydroepiandrosterone sulfate (DHEAS) has known anti-glucocorticoid effects and may counter the effects of cortisol.

**Objectives:**

We aimed to examine whether CSF cortisol and DHEAS levels were associated with (1) neuropsychiatric symptoms at baseline, (2) changes in neuropsychiatric symptoms over 3 years, and (3) whether these associations were related to or independent of AD pathology.

**Methods:**

One hundred and eighteen participants on a prospective study in a memory clinic setting, including patients with cognitive impairment (*n* = 78), i.e., mild cognitive impairment or mild dementia, and volunteers with normal cognition (*n* = 40), were included. Neuropsychiatric symptoms were assessed using the Neuropsychiatric Inventory Questionnaire (NPI-Q). CSF cortisol and DHEAS, as well as CSF AD biomarkers, were obtained at baseline. Neuropsychiatric symptoms were re-assessed at follow-up visits 18 and 36 months from baseline. We constructed linear regression models to examine the links between baseline neuropsychiatric symptoms, the presence of AD pathology as indicated by CSF biomarkers, and CSF cortisol and DHEAS. We used repeated-measures mixed ANCOVA models to examine the associations between the neuropsychiatric symptoms’ changes over time, baseline CSF cortisol and DHEAS, and AD pathology.

**Results:**

Higher CSF cortisol was associated with higher NPI-Q severity scores at baseline after controlling for covariates including AD pathology status (*B* = 0.085 [0.027; 0.144], *p* = 0.027; *r* = 0.277). In particular, higher CSF cortisol was associated with higher baseline scores of depression/dysphoria, anxiety, and apathy/indifference. Elevated CSF cortisol was also associated with more marked increase in NPI-Q scores over time regardless of AD status (*p* = 0.036, *η*^2^ = 0.207), but this association was no longer significant after controlling for BMI and the use of psychotropic medications. CSF DHEAS was associated neither with NPI-Q scores at baseline nor with their change over time. Cortisol did not mediate the association between baseline NPI-Q and changes in clinical dementia rating sum of boxes over 36 months.

**Conclusion:**

Higher CSF cortisol may reflect or contribute to more severe neuropsychiatric symptoms at baseline, as well as more pronounced worsening over 3 years, independently of the presence of AD pathology. Our findings also suggest that interventions targeting the HPA axis may be helpful to treat neuropsychiatric symptoms in patients with dementia.

**Supplementary Information:**

The online version contains supplementary material available at 10.1186/s13195-022-01139-9.

## Introduction

Neuropsychiatric symptoms (NPS) are increasingly recognized as core symptoms in dementia and Alzheimer’s disease (AD) [1, 2]. While these symptoms are very common at advanced dementia stages, they can also occur at pre-dementia stages and even precede the onset of cognitive symptoms [3, 4].

NPS have been linked to poorer quality of life and to more pronounced impairment in activities of daily living [1, 4]. Their presence has also been associated with faster disease progression and earlier institutionalization [2, 5]. NPS, especially irritability, agitation, sleep disturbances, anxiety, apathy, and delusion, have also been shown to increase the caregiver burden [6]. Yet, these symptoms are often under-recognized and inadequately managed [2]. The better understanding of the underlying pathophysiological mechanisms and the identification of potential biomarkers may help improve detection and targeted interventions at very early stages, when pharmacological and non-pharmacological interventions might be more effective [7–9].

Pathophysiology of NPS in dementia and AD remains poorly understood: while there is evidence linking these symptoms to Aβ pathology, the relationships with tau pathology and neurodegeneration seem to be less clear [8]. Cortisol may have an important role in the pathophysiology of NPS, in cognitive decline and AD. Indeed, elevated cortisol has been consistently associated with poorer cognitive functioning, neurodegeneration, more severe AD pathology, and faster disease progression [10–12]. There is also substantial evidence that elevated cortisol is associated with several neuropsychiatric symptoms, including mood and psychosis symptoms, and delirium [13, 14]. Dehydroepiandrosterone sulfate (DHEAS) is known to exert anti-glucocorticoid effects on the brain and may also be involved in the pathophysiology of NPS in dementia and AD [15]. DHEAS could also have positive effects on cognition and to improve behavioral symptoms [16], although previous findings were inconsistent [5].

It is possible that elevated cortisol levels and cortisol/DHEAS ratio can reflect and/or contribute to more pronounced NPS in older people not only through worsening AD pathology and neurodegeneration [12], but possibly also through other mechanisms that are independent of the core AD pathology [17].

In the present study, we aimed to examine whether the CSF levels of cortisol and DHEAS and the cortisol/DHEAS ratio were associated with (1) NPS at baseline, (2) changes in NPS over 3 years, and (3) whether these associations were related to or independent of AD pathology. We hypothesize that elevated CSF cortisol and CSF cortisol/DHEAS ratio would be associated with more severe NPS at baseline and with more pronounced worsening of NPS over time, even after controlling for the presence of AD pathology as indicated by CSF biomarkers. Considering that higher cortisol has been associated with faster cognitive decline, we additionally explored whether cortisol may mediate the association between NPS and cognitive decline at follow-up visits.

## Methods

### Participants

We recruited 118 community-dwelling subjects aged between 49 and 88 years in an observational study on biomarkers of cognitive decline and AD conducted at the Department of Psychiatry and the Department of Clinical Neurosciences, University Hospital of Lausanne, Switzerland.

The study participants with cognitive impairment (*n*=78) were recruited among outpatients referred to the Memory Clinics, Department of Psychiatry and the Department of Clinical Neurosciences, University Hospital of Lausanne, and who were diagnosed with MCI [18] or mild dementia according to published criteria [18, 19] based on neuropsychological and clinical evaluations followed by a consensus conference of senior physicians and neuropsychologists. All subjects in this group had a Clinical Dementia Rating (CDR) scale [20] score ≥ 0.5 based on the clinical and neuropsychological examination and considering informant questionnaires on the patient’s health and activities of daily living, as described elsewhere [21]. Participants with a diagnosis of MCI or mild dementia were merged into one group, consistently with the concept that both clinical stages are part of a clinical continuum of developing dementia and AD [22]. Cognitively healthy individuals (*n*=40) were recruited through journal announcements and word of mouth. They had the same clinical and neuropsychological examination as the cognitively impaired participants. They had no history or clinical signs of cognitive decline and a CDR=0. Subjects with any concomitant neurological, psychiatric, or somatic comorbidity or current medication that could affect cognition at baseline were not included. Patients on corticosteroids were excluded. However, patients on psychotropic medications were not excluded if the medication regimen was deemed stable and not interfering with cognition in the clinician’s opinion. The intake of antidepressants, antipsychotics, benzodiazepines, antiepileptics, and anti-dementia drugs has been systematically recorded.

At follow-up visits 18 and 36 months from baseline, cognitive and functional performance was assessed using the same methods as at baseline.

### Cognitive assessment

We assessed the global cognitive performance using the Mini-Mental State Examination (MMSE).

We also used a battery of cognitive tests to assess the most important cognitive domains (including episodic memory, verbal fluency, executive functions, and visuospatial construction) and proxy questionnaires to evaluate basic and instrumental activities of daily living, as previously described [5]. These tools are validated and widely used in the field. The results were used to evaluate the cognitive and functional status using the CDR scale, a scale that is widely used for the clinical staging of cognitive impairment [20]. We calculated the CDR sum of boxes score (CDR-SB) as a measure of clinical disease severity.

### Assessment of neuropsychiatric symptoms

We assessed NPS using the Neuropsychiatric Inventory Questionnaire (NPI-Q) in its validated French version [23]. The NPI-Q is a self-administered questionnaire which can be filled by caregivers of patients with dementia. It evaluates 12 different behavioral and psychological symptoms in patients with dementia (delusions, hallucinations, dysphoria, anxiety, agitation/aggression, euphoria, disinhibition, irritability/lability, apathy, aberrant motor activity, sleep disturbances, and appetite disturbances) [24]. For each reported symptom, the NPI-Q provides a severity score (on a 3-point scale) and a caregiver distress score (on a 5-point scale), with higher scores indicating more severe psychopathology. We calculated the NPI-Q total severity score by summing up the 12 severity subscores, and the total caregiver distress score by summing up the 12 caregiver distress subscores [24, 25]. NPI-Q was administered at baseline and the follow-up visits 18 and 36 months from baseline.

The time between CSF sampling and NPI-Q questionnaire administration was required to be less than 6 weeks. The proxies were asked to state the situation at the time point of the CSF sampling.

### Cerebrospinal fluid biomarkers of AD pathology and cortisol and DHEAS levels

We collected 10–12 mL of CSF by lumbar puncture between 8 and 9 AM after overnight fasting. The CSF samples were spun down at 4°C, immediately aliquoted, snap frozen at −80 °C, and stored at −80 °C until assay, with no thawing before analysis [26]. The first 2–4mL was used for routine analysis, but no specific order of the remaining samples was considered. CSF samples were stored for up to 5 years.

CSF amyloid-β_1-42_ (Aβ_1-42_), tau, and tau phosphorylated at threonine 181 (p-tau181) were measured using commercially available enzyme-linked immunosorbent assay kits (Fujirebio, Gent, Belgium).

We used the CSF p-tau181/Aβ_1-42_ ratio with a cut-off of 0.0779 to define the presence/absence of an AD CSF profile (indicating the presence/absence of cerebral AD pathology), previously determined as the center cutoff indicating the concomitant presence of amyloid and tau pathology [26].

CSF cortisol and DHEAS concentrations were measured using commercially available chemiluminescence immunoassay with high sensitivity (IBL International, Hamburg, Germany). We measured DHEAS rather than DHEA in the present study because DHEAS was shown to be a robust indicator of DHEA production [27]. The intra- and interassay coefficients for cortisol and DHEAS were below 6% and 9%, respectively.

### Apolipoprotein E genotyping

The *apolipoprotein E (APOE)* genotype was determined as previously described [21]. We split participants into two groups: APOEε4 carriers (carrying at least one APOEε4 allele) and APOEε4 non-carriers.

### Standard protocol approvals, registrations, and patient consents

The study was conducted in accordance with applicable laws and regulations, including the International Conference on Harmonization, Guideline for Good Clinical Practice, and the ethical principles that have their origins in the Declaration of Helsinki [28]. The local ethical committee approved this study (No. 171/2013), and all participants or their legal representatives provided written informed consent.

### Statistical analysis

Statistical analysis was performed using SPSS v26.0 (IBM Corp., Armonk, NY, USA).

#### Descriptive statistics

We determined absolute and relative frequencies for categorical variables. We calculated means and standard deviations for continuous variables (in case of normality as per the Shapiro-Wilk test) or the median and interquartile range (IQR) in case of non-normality.

We also used Spearman’s correlations to examine the associations between baseline NPI-Q scores and changes in CDR-SB over 18 and 36 months, controlling for age, sex, and APOEε4 status.

#### Associations between baseline neuropsychiatric symptoms and cerebrospinal fluid cortisol and dehydroepiandrosterone sulfate

We used the independent samples *t*-test to compare CSF cortisol, CSF DHEAS, and CSF cortisol/DHEAS ratio between participants with baseline NPS (NPI-Q>0) and those without (NPI-Q=0).

We constructed a multiple linear regression model to examine the links between baseline NPI-Q total severity scores, AD pathology, and CSF cortisol and DHEAS, controlling for APOEε4 status, sex, and age.

To determine which neuropsychiatric subsyndromes are associated with CSF cortisol in particular, we used a multivariate analysis of covariance (MANCOVA) with the twelve NPI-Q severity subscores as dependent variables and with CSF cortisol, CSF DHEAS, CSF AD pathology, APOEε4 status, sex, and age as independent variables. It is worth mentioning that APOEε4 status was added to the model because APOEε4 significantly differed between participants with and those without NPS.

Preliminary assumptions for MANCOVA (including normality, linearity, univariate and multivariate outliers, covariance matrices, and multicollinearity) were tested. Pillai’s trace test was used because the NPI-Q scores violated the normality assumption. The effect size was assessed using the partial eta squared (*η*^2^).

#### Associations between cerebrospinal fluid cortisol and dehydroepiandrosterone sulfate and changes in neuropsychiatric symptoms over 36 months

We used repeated-measures mixed analysis of covariance (ANCOVA) models with:

- The NPI-Q total severity scores (at baseline, at 18 months, and at 36 months) as within-subject variables
- With sex, AD pathology status, and APOEε4 status as between-subject factors
- With age, CSF cortisol, and CSF DHEAS as covariates

Global analyses were followed by between-subject and within-subject analyses to highlight significant interactions. The Greenhouse-Geisser correction was applied whenever the sphericity assumption was not fulfilled. Effect sizes were determined using partial eta squares (*η*^2^).

#### Cerebrospinal fluid cortisol as a potential mediator of the association between baseline NPS and cognitive and functional decline

We conducted Sobel’s test for mediation using the regression between CSF cortisol and neuropsychiatric symptoms at baseline (controlling for age, sex, AD pathology, and APOEε4 status) and the regression between the changes in CDR-SB and CSF cortisol (controlling for age, sex, AD pathology, and APOEε4 status).

For each of the repeated-measures ANCOVA and regression models above, we also constructed models adding psychotropic medications and body mass index (BMI) to covariates. Indeed, previous research indicated that psychotropic medications including antidepressants, antipsychotics, and stimulants can affect cortisol levels [29]. Similarly, cortisol levels were shown to depend on BMI [21].

For all statistical tests, the alpha value was set at 0.05. For multiple comparisons, *p* values were adjusted according to Holm-Bonferroni’s method.

## Results

### Baseline characteristics

Out of the 118 participants included, NPI-Q was available for *n*=100 subjects at baseline, *n*=75 at 18 months, and *n*=39 at 36 months. Baseline CSF cortisol and DHEAS as well as baseline cognitive assessment were available for all participants. CDR and MMSE were available in *n*=103 individuals at 18 months and in *n*=96 individuals at 36 months. The first follow-up actually took place at 17.8±2.7 months. The second follow-up was conducted at 35.5±3.5 months.

Baseline sample characteristics of all subjects and subgroups defined according to baseline NPS are given in Table 1. Even though 18 (15.3%) participants did not have a baseline NPI-Q, these were still included in other analyses.

**Table 1:** General characteristics of the study participants at baseline

	Total sample	Participants without baseline NPS	Participants with baseline NPS	***p***
***n***^a^	118	44	56	
**Age, years** (m ±SD)	71.2±8.2	69.0±8.6	72.1±6.9	0.049
**Sex,*n*(%) women**	70 (59.3%)	17 (38.6%)	23 (41.1%)	0.840
**Education level,*n*(%) higher education**	35 (30.2%)	18 (41.9%)	16 (29.1%)	0.188
**APOEε4,*n*(%) carriers**	40 (36.0%)	7 (16.3%)	25 (50.0%)	0.001
**CDR-SB (0–18)** (m ±SD)	1.5±2.1	0.7±1.4	2.2±2.4	<0.001
**MMSE score (0–30)** (m ±SD)	26.3±3.6	27.4±3.8	25.4±3.6	0.010
**Psychotropic drugs,*n*(%)**	29 (25%)	4 (9.1%)	20 (36.4%)	0.002
**BMI, kg/m**^**2**^ (m ±SD)	25.0±4.2	24.6±3.6	25.4±4.9	0.388
**CSF AD biomarkers**	800.9±281.2			
Aβ_1-42_, in pg/mL (m±SD)	423.1±322.0	876.7±267.7	725.5±276.4	0.007
tau, in pg/mL (m±SD)	65.5±38.3	285.9.1±198.6	511.2±366.9	<0.001
p-tau181, in pg/mL (m±SD)		51.3±23.0	75.2.9±46.2	0.002
**CSF AD pathology status**^b^**,*n*(%)**	51 (43.2%)	8 (18.2%)	32 (57.1%)	<0.001
**CSF cortisol**, in ng/mL (m ±SD)	36.77±17.21	29.6±12.7	41.0±18.6	<0.001
**CSF DHEAS**, in ng/mL (m ±SD)	1.01±0.45	1.02±0.457	0.942±0.421	0.376
**CSF cortisol/DHEAS** ratio (m ±SD)	45.63±36.84	35.7±26.8	55.0±44.1	0.012

*AD* Alzheimer’s disease, *APOE* apolipoprotein E, *BMI* body mass index, *CDR* Clinical Dementia Rating, *CDR-SB* Clinical Dementia Rating Sum of Boxes, *CI* cognitive impairment, *CSF* cerebrospinal fluid, *DHEAS* dehydroepiandrosterone sulfate, *m* mean, *MMSE* Mini-Mental State Examination, *NPS* neuropsychiatric symptoms, *p-tau181* hyperphosphorylated tau, *SD* standard deviation, *tau* total tau

^a^Baseline NPI-Q was not available for 18 (15.3%) participants

^b^CSF AD pathology status was defined with p-tau181/Aβ1-42 ratio ≥ 0.0779

Participants with NPS at baseline (*n*=56) were older and more likely to be APOEε4 carriers than participants without (*n*=44). No differences were found between groups regarding sex or education level (Table 1).

The most common NPS at baseline were anxiety (37%, *n*=37), apathy/indifference (30%, *n*=30), depression/dysphoria (28%, *n*=28), and irritability/lability (21%, *n*=21) (Supplementary Table 1).

Supplementary Table 2 shows the distribution of participants according to the AT(N) system classification of AD pathology [30].

We also found positive correlations between baseline NPI-Q and changes in CDR-SB at 18 months (rho=0.285, Holm-Bonferroni-adjusted *p*=0.022) and 36 months (rho=0.371, Holm-Bonferroni-adjusted *p*≤0.001), controlling for age, sex, and APOEε4 status.

When comparing participants with baseline cognitive impairment (CDR≥0.5) to those without (CDR=0), we found a higher prevalence of baseline NPS in the cognitive impairment group (68.8% vs 33.3%, *p*<0.001).

### Associations between baseline neuropsychiatric symptoms and cerebrospinal fluid cortisol and dehydroepiandrosterone sulfate

**Fig. 1:**
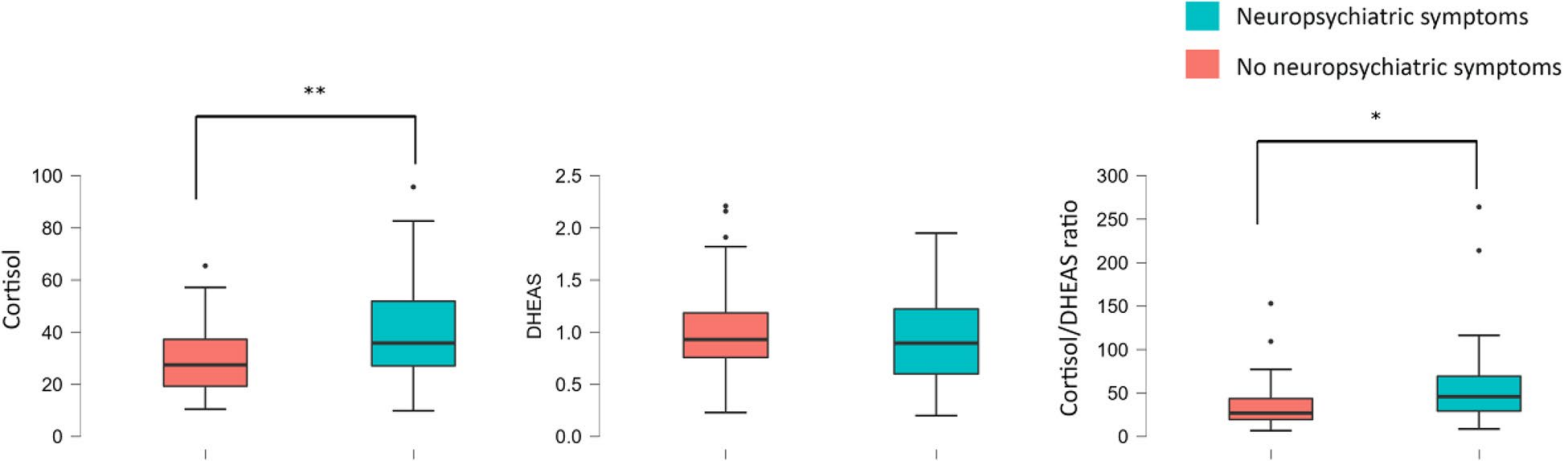
Boxplots of cerebrospinal fluid cortisol and dehydroepiandrosterone sulfate levels in participants with/without neuropsychiatric symptoms at baseline.*p=0.024. **p<0.001. Cerebrospinal fluid cortisol and cortisol/DHEAS ratio were significantly higher in subjects with neuropsychiatric symptoms at baseline. Associations shown in this figure were not adjusted for covariates. *p* values were adjusted for multiple comparisons using Holm-Bonferroni’s method

Participants with NPS at baseline had higher CSF cortisol (40.95±18.63 vs 29.60±12.73, Holm-Bonferroni-adjusted *p*<0.001) and higher CSF cortisol/DHEAS ratio (55.00±44.08 vs 35.66±26.83, Holm-Bonferroni-adjusted *p*=0.024) than participants without. CSF DHEAS did not differ significantly between groups (Table 1 and Fig. 1).

More severe neuropsychiatric symptoms at baseline were associated with higher CSF cortisol but not with CSF DHEAS, after controlling for sex, age, APOEε4 status, and AD pathology status. The presence of AD pathology was also associated with more severe neuropsychiatric symptoms. When baseline cognitive impairment was added to the model, it did not have any association with the NPI-Q severity score and it did not alter the results. When adding the use of psychotropic medications and BMI as covariates, we found that the use of psychotropic drugs was significantly associated with the NPI-Q severity score (*p*=.009). Even though the association between CSF cortisol and the NPI-Q severity score became “weaker,” it remained statistically significant (Table 2).

**Table 2:** Multiple linear regression model with the severity of neuropsychiatric symptoms as a dependent variable and cerebrospinal fluid cortisol and dehydroepiandrosterone sulfate as independent variables

*Model 1: Independent variables: CSF cortisol, CSF DHEAS, CSF AD pathology, sex, age, APOEε4 status*						
			**95.0% confidence interval for*B***		**Partial regression**	
**Variable**	***B***	**Sig.**	**Lower bound**	**Upper bound**		**VIF**
**CSF cortisol**	.085	.005	.027	.144	.277	1.189
**CSF DHEAS**	−1.060	.365	−3.370	1.251	−.086	1.123
**CSF AD pathology**	2.765	.022	.414	5.117	.222	1.129
**Sex**	.508	.599	−1.406	2.421	.050	1.307
**Age**	−.009	.893	−.141	.123	−.013	1.686
**APOEε4 status**	.213	.853	−2.062	2.488	.018	1.517
*Model 2: Independent variables: CSF cortisol, CSF DHEAS, CSF AD pathology, sex, age, APOEε4, BMI, use of psychotropic medication*						
			**95.0% confidence interval for*B***		**Partial regression**	
**Variable**	***B***	**Sig.**	**Lower bound**	**Upper bound**		**VIF**
**CSF cortisol**	.080	.008	.021	.139	.283	1.216
**CSF DHEAS**	−1.133	.332	−3.443	1.176	−.105	1.126
**CSF AD pathology**	2.225	.083	−.296	4.746	.187	1.945
**Sex**	.499	.605	−1.411	2.409	.056	1.129
**Age**	−.045	.541	−.190	.101	−.066	1.589
**APOEε4 status**	.074	.949	−2.209	2.357	.007	1.534
**Baseline cognitive impairment**	1.468	.246	−1.032	3.968	.126	1.913
*Model3: Independent variables: CSF cortisol, CSF DHEAS, CSF AD pathology, sex, age, APOEε4, BMI, use of psychotropic medication*						
			**95.0% confidence interval for*B***		**Partial regression**	
**Variable**	***B***	**Sig.**	**Lower bound**	**Upper bound**		**VIF**
**CSF cortisol**	.062	.039	.003	.121	.225	1.325
**CSF DHEAS**	−1.343	.238	−3.590	.904	−.129	1.148
**CSF AD pathology**	3.779	.002	1.377	6.181	.325	1.883
**Sex**	1.200	.213	−.703	3.103	.136	1.209
**Age**	−.060	.365	−.191	.071	−.099	1.405
**APOEε4 status**	−.407	.719	−2.645	1.831	−.040	1.568
**BMI**	.137	.202	−.075	.349	.140	1.221
**Use of psychotropic medication**	2.958	.009	.757	5.159	.281	1.273

*AD* Alzheimer’s disease, *APOE* the apolipoprotein E, *BMI* body mass index, *CSF* cerebrospinal fluid, *DHEAS* dehydroepiandrosterone sulfate, *VIF* variance inflation factor

Higher CSF cortisol was in particular associated with higher scores in the following neuropsychiatric symptoms: depression/dysphoria (*p*=0.003; *η*^2^=0.103), anxiety (*p*=0.001; *η*^2^=0.134), and apathy/indifference (*p*=0.001; *η*^2^=0.139) (Table 3).

**Table 3:** Multivariate analysis of covariance (MANCOVA) with the twelve Neuropsychiatric Inventory severity subscores as dependent variables and with CSF cortisol, CSF DHEAS, Alzheimer’s disease CSF pathology, APOE status, sex, and age as independent variables

**Effect**		**Pillai’s trace**	***F***	**Error df**	**Sig.**	**Partial eta squared**
**Intercept**		.078	.481^b^	68.000	.919	.078
**CSF cortisol**		**.257**	**1.962**^**b**^	**68.000**	**.042**	**.257**
**DHEAS**		.111	.705^b^	68.000	.741	.111
**Age**		.081	.501^b^	68.000	.907	.081
**Sex**		.284	2.249^b^	68.000	.018	.284
**AD pathology status**		**.342**	**2.951**^**b**^	**68.000**	**.002**	**.342**
**APOEε4 status**		.219	1.588^b^	68.000	.116	.219
Sex * CSF AD pathology		.233	1.723^b^	68.000	.081	.233
Sex * APOEε4 status		.229	1.682^b^	68.000	.090	.229
CSF AD pathology * APOEε4 status		.219	1.585^b^	68.000	.117	.219
Sex * CSF AD pathology * APOEε4 status		.196	1.377^b^	68.000	.198	.196
**Tests of between-subject effects**						
**Variable**	**Dependent variable**	**Type III sum of squares**	**Mean square**	***F***	**Sig.**	**Partial eta squared**
**CSF cortisol**	Delusions	.027	.027	.235	.630	.003
	Hallucinations	.037	.037	.795	.375	.010
	Agitation/aggression	.092	.092	.202	.654	.003
	**Depression/dysphoria**	**5.691**	**5.691**	**9.093**	**.003**	**.103**
	**Anxiety**	**8.783**	**8.783**	**12.210**	**.001**	**.134**
	Elation/euphoria	.054	.054	.600	.441	.008
	**Apathy/indifference**	**9.300**	**9.300**	**12.731**	**.001**	**.139**
	Disinhibition	.087	.087	.594	.443	.007
	Irritability/lability	1.599	1.599	3.105	.082	.038
	Motor disturbance	.213	.213	1.257	.266	.016
	Nightime behaviors	.871	.871	2.363	.128	.029
	Appetite/eating	.739	.739	1.830	.180	.023
**AD pathology**	Delusions	.315	.315	2.698	.104	.033
	Hallucinations	.049	.049	1.050	.309	.013
	**Agitation/aggression**	**2.085**	**2.085**	**4.586**	**.035**	**.055**
	Depression/dysphoria	.809	.809	1.293	.259	.016
	Anxiety	2.297	2.297	3.194	.078	.039
	Elation/euphoria	.039	.039	.430	.514	.005
	**Apathy/indifference**	**4.911**	**4.911**	**6.722**	**.011**	**.078**
	Disinhibition	.336	.336	2.283	.135	.028
	Irritability/lability	1.426	1.426	2.769	.100	.034
	**Motor disturbance**	**.910**	**.910**	**5.366**	**.023**	**.064**
	Nighttime behaviors	.014	.014	.038	.846	.000
	Appetite/eating	.002	.002	.005	.945	.000

*AD* Alzheimer’s disease, *APOE* the apolipoprotein E, *CSF* cerebrospinal fluid, *DHEAS* dehydroepiandrosterone sulfate

### Associations between cerebrospinal fluid cortisol, dehydroepiandrosterone sulfate, AD pathology status, and changes in neuropsychiatric symptoms over 36 months

Mixed design repeated-measures ANCOVAs found significant interactions between NPI-Q total severity score changes 36 months after baseline and CSF cortisol (*p*=0.036, *η*^2^=0.207) but not with CSF DHEAS. Higher CSF cortisol predicted more pronounced worsening in NPS over 3 years. However, when BMI and the use of psychotropic medications were added to the model, this association was no longer significant (*p*=0.268, *η*^2^=0.103).

The presence of AD pathology did not predict longitudinal changes in NPS (Table 4).

**Table 4:** Repeated-measures mixed analysis of covariance (ANCOVA) with the Neuropsychiatric Inventory total severity scores over 36 months as a within-subject variable and with CSF cortisol, CSF DHEAS, Alzheimer’s disease CSF pathology, APOE status, sex, and age as independent variables

*Model 1: Independent variables: CSF cortisol, CSF DHEAS, Alzheimer’s disease CSF pathology, APOE status, sex, and age*					
**Variable**	**Type III sum of squares**	**Mean square**	***F***	**Sig.**	**Partial eta squared**
**Time**	4.418	3.310	.905	.380	.051
Time * age	5.767	4.321	1.181	.306	.065
Time * CSF cortisol	**21.713**	**16.269**	**4.447**	**.036**	**.207**
Time * CSF DHEAS	12.085	9.055	2.475	.122	.127
Time * sex	8.921	6.685	1.827	.190	.097
Time * AD pathology status	.000	.	.	.	.000
Time * APOEε4	10.107	7.573	2.070	.160	.109
Time * sex * AD pathology	.000	.	.	.	.
Time * sex * APOEε4	.000	.	.	.	.
Time * AD pathology * APOEε4	.000	.	.	.	.
Time * sex * AD pathology * APOEε4	.000	.	.	.	.
*Model 2: Independent variables: CSF cortisol, CSF DHEAS, Alzheimer’s disease CSF pathology, APOE status, sex, age, BMI, and use of psychotropic medication*					
**Variable**	**Type III sum of squares**	**Mean square**	***F***	**Sig.**	**Partial eta squared**
**Time**	2.887	2.350	.853	.394	.066
Time * age	3.660	2.979	1.081	.331	.083
Time * CSF cortisol	4.662	3.795	1.377	.268	.103
Time * CSF DHEAS	5.612	4.568	1.657	.222	.121
Time * BMI	.017	.014	.005	.968	.000
Time * sex	12.179	9.915	3.597	.071	.231
Time * AD pathology	.000	.	.	.	.
Time * APOEε4	11.327	9.221	3.346	.081	.218
Time * use of psychotropic medication	8.058	6.560	2.380	.141	.166
Time * sex * AD pathology	.000	.	.	.	.
Time * sex * APOEε4	.000	.	.	.	.
Time * sex * use of psychotropic medication	4.306	3.506	1.272	.289	.096
Time * AD pathology * APOEε4	.000	.	.	.	.
Time * AD pathology * use of psychotropic medication	.000	.	.	.	.
Time * APOEε4 * use of psychotropic medication	7.218	5.876	2.132	.164	.151
Time * sex * AD pathology * APOEε4	.000	.	.	.	.
Time * sex * AD pathology * use of psychotropic medication	.000	.	.	.	.
Time * sex * APOEε4 * use of psychotropic medication	.000	.	.	.	.
Time * AD pathology * APOEε4 * use of psychotropic medication	.000	.	.	.	.
Time * sex * AD pathology * APOEε4 * use of psychotropic medication	.000	.	.	.	.

*AD* Alzheimer’s disease, *APOE* the apolipoprotein E, *BMI* body mass index, *CSF* cerebrospinal fluid, *DHEAS* dehydroepiandrosterone sulfate, *NPI-Q* Neuropsychiatric Inventory Questionnaire

Sobel’s test did not show that cortisol mediated the association between baseline NPI-Q total severity score and CDR-SB changes over 18 or over 36 months (Supplementary Table 3).

## Discussion

In this prospective study, we found that higher CSF cortisol was associated with higher NPI-Q scores indicating more severe baseline NPS in general and the following domains in particular: depression/dysphoria, anxiety, and apathy/indifference. While the presence of AD pathology as indicated by CSF biomarkers was also associated with NPS, CSF cortisol levels were associated with the severity of NPS independently of AD. Higher baseline CSF cortisol was associated with more pronounced worsening in NPS over 3 years, controlling for age, sex, AD pathology, and APOEε4 status. CSF DHEAS was associated neither with NPS at baseline nor with their change over time.

### Associations between baseline neuropsychiatric symptoms and cerebrospinal fluid cortisol dehydroepiandrosterone sulfate

We found that higher CSF cortisol was cross-sectionally associated with more severe NPS in general, and with depression/dysphoria, anxiety, and apathy/indifference in particular, independently of the presence of AD pathology. A relationship between cortisol and NPS in patients with dementia has been previously suggested. Indeed, a previous study has found that a flatter diurnal salivary cortisol slope was linked to agitation and disinhibition, while a steeper slope was associated with anxiety and insomnia [31]. Another study linked higher serum cortisol to increased dysphoria, but not to anxiety or depressive symptoms in patients with mild to moderate AD dementia [32]. An environmental intervention (interaction with the natural environment in an indoor therapeutic garden) that was shown to improve NPS in patients with clinically diagnosed AD also decreased their salivary cortisol levels. Authors hypothesized that the intervention decreased the abnormally high cortisol levels in patients, thus improving their NPS and their cognition [33].

Consistent with our findings, higher cortisol has been previously associated with anxiety, depression, irritability, and apathy in individuals with psychiatric disorders, and cortisol is considered one of the most significant biomarkers for depressive and anxiety disorders [14, 34].

The exact mechanisms that may explain the relationship between high cortisol and NPS are complex. There has been substantial research on the role of the HPA axis in many psychiatric disorders over the life span, and cortisol has been involved in the pathogenesis of most of the psychiatric symptoms [35–38]. It is likely that the more pronounced AD pathology observed with high cortisol can increase the likelihood of NPS, since NPS tend to be more frequent at more advanced stages of AD [3]. It is also possible that occurring depression with increased cortisol can contribute to cognitive decline, not only through contributing to AD pathology, but also through direct actions of chronically high cortisol on brain structures like the hippocampus and the prefrontal cortex [12, 39]. Of note, we observed associations of CSF cortisol levels with NPS independent of the presence of AD pathology, suggesting that increased cortisol in the CNS is involved in the pathogenesis of NPS through mechanisms that are at least in part independent of AD.

To the best of our knowledge, there have been no previous studies directly investigating the links between DHEAS and NPS in patients with AD, and the results of previous studies about the effects of DHEAS on the brain have been inconsistent [16]. On the one hand, there is evidence that DHEAS can antagonize some of the deleterious effects of cortisol on the brain and that higher DHEAS might be associated with lower stress levels in patients with dementia [16, 40]. On the other hand, in a small-scale randomized, double-blind, placebo-controlled study of DHEA treatment in patients with AD, participants in the DHEA group were more likely to show anxiety, agitation, or paranoid symptoms than those in the placebo group [41]. Considering our results and the limited previous evidence, it remains difficult to draw any conclusions with regard to the associations between DHEAS and NPS in AD.

### Associations between cerebrospinal fluid cortisol and dehydroepiandrosterone sulfate, CSF AD pathology, and changes in neuropsychiatric symptoms over 36 months

Our findings suggest that elevated CSF cortisol, but not CSF DHEAS, may predict more pronounced worsening in NPS over 3 years independently of CSF AD pathology.

To the best of our knowledge, no previous studies investigated the links between cortisol and longitudinal overall changes in NPS. Nonetheless, a previous longitudinal study reported that higher baseline salivary cortisol predicted depressive symptoms 3 years later [42]. Our results support both the contribution of increased cortisol levels in the CNS to the pathogenesis of NPS persisting or worsening over time. Since NPS are increasingly recognized as a major determinant of prognosis in cognitive decline and AD, the early identification and treatment of factors that may contribute to their manifestation and persistence may translate into better outcomes [1]. The use of a biomarker that can predict the worsening of these symptoms can possibly lead to further tailoring of the management plan to each individual case, in the sense that a treatment intervention might be warranted if a significant deterioration is predicted. In addition, we may hypothesize that treatment modalities aiming to decrease elevated cortisol levels may improve the course of NPS over time. Indeed, environmental interventions [33] and aerobic exercise [43] in older people with dementia may reduce NPS severity and this effect may be due, at least in part, to reduced cortisol levels [12, 33]. There is also evidence that hypercortisolism-associated neuropsychiatric symptoms can subside after curative surgery in patients with endogenous Cushing’s syndrome [44]. Furthermore, certain HPA-targeting agents have been shown to improve depressive and psychotic symptoms in the adult population [45]. It remains to be seen, however, whether these interventions can also help improve NPS in older subjects with or without AD pathology.

This said, when we added the use of psychotropic medications to the model, the association between cortisol and longitudinal changes in NPS was no longer significant. It is likely that the use of psychotropic medications can improve the patients’ NPS even in those with higher CSF cortisol levels, thus masking the link between baseline cortisol and the course of NPS.

The relationship between DHEAS and longitudinal changes of NPS has not been previously investigated. The established effects of DHEAS on cortisol and the GABAergic, cholinergic, serotoninergic, norepinephrinergic, and dopaminergic systems in the brain can mean that DHEAS is probably involved in the pathophysiology of NPS [16]. In addition, DHEA was previously reported to exhibit some antidepressant, anxiolytic, and anti-aggressive effects [46]. The negative result in our study can be due to inter-individual differences regarding the complex factors that determine CSF DHEAS levels, including systemic levels of DHEAS, brain synthesis of DHEA(S), conversion from DHEA to DHEAS, and conversion of DHEA(S) into testosterone and estrogens, as well as cortisol levels [47]. Our results suggest that CSF DHEAS levels do not represent a useful marker to predict the course of NPS.

### Limitations and strengths

One limitation of this study is that CSF cortisol and DHEAS levels were solely measured at baseline. In addition, while the first 2–4mL of CSF was used for routine analysis, no specific order of the remaining samples was considered. Therefore, we could not exclude some differences due to the order of the samples. Nonetheless, previous studies do not suggest the existence of a CSF gradient for cortisol [48].

One further limitation is related to the sample size with relatively small numbers of single neuropsychiatric symptoms. For symptoms such as delusions, hallucinations, elation/euphoria, disinhibition, and motor disturbance, the subgroups were too small to allow for detecting potential associations. However, single neuropsychiatric symptoms may have at least in part distinct pathological underlying processes [49]. Also, for the definition of AD pathology, we chose a binary classification (presence/absence) using CSF p-tau181/Aβ1-42 as a marker of the concomitant presence of amyloid pathology and tau pathology [50–52] because of the relatively small sample size, not allowing for a more detailed classification such as the AT(N) [53].

Furthermore, we have included cognitively unimpaired subjects and memory clinic patients with MCI and mild dementia, while patients with either more severe dementia stages or with severe psychiatric syndromes that may interfere with cognitive performance were not included. Accordingly, our results may not be fully representative for the elderly population, and in particular for advanced dementia. However, applying these selection criteria may still have led to a heterogeneous sample, reflecting a part of the heterogeneity in the memory clinic setting.

However, including and following up cognitively healthy participants and patients at early stages of cognitive decline is an important strength of our work. It allows for investigating at a very early stage the putative influence of cortisol and DHEAS levels on the further course of the disease. This study is, to the best of our knowledge, the first prospective study to investigate the relationship between CSF cortisol and DHEAS, and NPS severity, and their changes over the years. We also measured central nervous system rather than peripheral steroid (cortisol and DHEAS) levels, to reflect more accurately the levels in the brain [54, 55].

A further strength is that CSF biomarkers of AD pathology were measured in all participants allowing to address the relationships of the observed associations with the presence or absence of AD.

## Conclusions

Our findings suggest that elevated CSF cortisol may be linked to both more severe NPS and more pronounced worsening of NPS over the years. These relationships are probably not merely explained by the previously described effects of cortisol on AD pathology and neurodegeneration [12]. It is possible that they involve distinct and direct actions of cortisol on the brain, although this needs to be confirmed by future studies.

CSF cortisol might be a useful biomarker to predict the course of NPS over time, while interventions targeting the HPA axis might be useful in reducing NPS severity and preventing their presence or worsening over time. Further studies are needed to confirm these hypotheses.

## Supplementary Information

**Additional file 1: Supplementary Table 1.** Frequency of neuropsychiatric syndromes at baseline.

**Additional file 2: Supplementary Table 2.** Distribution of participants according to the AT(N) system classification of Alzheimer’s disease pathology.

**Additional file 3: Supplementary Table 3.** Sobel’s test regressions coefficients between cortisol and baseline neuropsychiatric symptoms, and between changes in CDR-SB and CSF cortisol.

## Data Availability

Anonymized data not published within this article will be made available by request from any qualified investigator.
