# Supplementary material for: Persisting neuropsychiatric symptoms, Alzheimer’s disease, and cerebrospinal fluid cortisol and dehydroepiandrosterone sulfate
Source: Alzheimers Res Ther. 2022 Dec 19;14:190. doi: 10.1186/s13195-022-01139-9 (PMC9762003; doi:10.1186/s13195-022-01139-9)
Supplement: Supplementary file 1 — Additional file 1: Supplementary Table 1. Frequency of neuropsychiatric syndromes at baseline. [file 13195_2022_1139_MOESM1_ESM.docx]

**Supplementary Table 1: Frequency of neuropsychiatric syndromes at baseline**

|  | **At baseline, n(%)** |
| --- | --- |
| **Delusions** | 5(5.0%) |
| **Hallucinations** | 2(2.0%) |
| **Agitation/Aggression** | 18(18.0%) |
| **Depression/Dysphoria** | 28(28.0%) |
| **Anxiety** | 37(37.0%) |
| **Elation/Euphoria** | 5(5.0%) |
| **Apathy/Indifference** | 30(30.0%) |
| **Disinhibition** | 4(4.0%) |
| **Irritability/Lability** | 21(21.0%) |
| **Motor Disturbance** | 7(7.0%) |
| **Nighttime Behaviors** | 17(17.0%) |
| **Appetite/Eating** | 16(16.0%) |
