# Supplementary material for: Persisting neuropsychiatric symptoms, Alzheimer’s disease, and cerebrospinal fluid cortisol and dehydroepiandrosterone sulfate
Source: Alzheimers Res Ther. 2022 Dec 19;14:190. doi: 10.1186/s13195-022-01139-9 (PMC9762003; doi:10.1186/s13195-022-01139-9)
Supplement: Supplementary file 2 — Additional file 2: Supplementary Table 2. Distribution of participants according to the AT(N) system classification of Alzheimer’s disease pathology. [file 13195_2022_1139_MOESM2_ESM.docx]

**Supplementary Table 2: Distribution of participants according to the AT(N) system classification of Alzheimer’s disease pathology**

| **A+T-N-** | 7(5.9%) |
| --- | --- |
| **A+T+N-** | 8(6.8%) |
| **A+T-N+** | 0(0.0%) |
| **A+T+N+** | 10(8.5%) |
| **A-T-N-** | 56(47.5%) |
| **A-T-N+** | 1(0.8%) |
| **A-T+N-** | 24(20.3%) |
| **A-T+N+** | 12(10.2%) |

*A+: Cerebrospinal fluid Amyloid-β_1-42_ < 550 pg/mL*

*T+: Cerebrospinal fluid tau phosphorylated at threonine 181 (p-tau181)>60 pg/mL*

*N+: Cerebrospinal fluid total tau >600 pg/mL*
