# Supplementary material for: Persisting neuropsychiatric symptoms, Alzheimer’s disease, and cerebrospinal fluid cortisol and dehydroepiandrosterone sulfate
Source: Alzheimers Res Ther. 2022 Dec 19;14:190. doi: 10.1186/s13195-022-01139-9 (PMC9762003; doi:10.1186/s13195-022-01139-9)
Supplement: Supplementary file 3 — Additional file 3: Supplementary Table 3. Sobel’s test regressions coefficients between cortisol and baseline neuropsychiatric symptoms, and between changes in CDR-SB and CSF cortisol. [file 13195_2022_1139_MOESM3_ESM.docx]

**Supplementary Table 3: Sobel’s test regressions coefficients between cortisol and baseline neuropsychiatric symptoms, and between changes in CDR-SB and CSF cortisol**

|  | | **Regression between CSF cortisol (dependent variable) and baseline neuropsychiatric symptoms** | | | | | | |
| --- | --- | --- | --- | --- | --- | --- | --- | --- |
|  | | |  |  |  | **95.0% Confidence Interval for B** | | **Partial regression** |
| **Variable** | | | **B** | **t** | **Sig.** | **Lower Bound** | **Upper Bound** |  |
|  | **Baseline total NPI-Q severity score** | | .991 | 2.706 | .008 | 0.263 | 1.719 | 0.285 |

|  | | **Regression between changes in CDR-SB over 18 months (dependent variable) and CSF cortisol** | | | | | | |
| --- | --- | --- | --- | --- | --- | --- | --- | --- |
|  | | |  |  |  | **95.0% Confidence Interval for B** | | **Partial regression** |
| **Variable** | | | **B** | **t** | **Sig.** | **Lower Bound** | **Upper Bound** |  |
|  | **CSF cortisol** | | .038 | 3.389 | .001 | .016 | .060 | .338 |

|  | | **Regression between changes in CDR-SB over 36 months (dependent variable) and CSF cortisol** | | | | | | |
| --- | --- | --- | --- | --- | --- | --- | --- | --- |
|  | | |  |  |  | **95.0% Confidence Interval for B** | | **Partial regression** |
| **Variable** | | | **B** | **t** | **Sig.** | **Lower Bound** | **Upper Bound** |  |
|  | **CSF cortisol** | | .040 | 2.225 | .029 | .004 | .076 | .232 |

**Sobel’s test statistics**

|  | **Sobel’s test statistic** | **Standard error** | **p value** |
| --- | --- | --- | --- |
| **CSF cortisol as a mediator of the association between baseline NPI-Q total severity score and CDR-SB changes over 18 months** | .011 | 3.360 | .991 |
| **CSF cortisol as a mediator of the association between baseline NPI-Q total severity score and CDR-SB changes over 36 months** | .018 | 2.208 | .986 |

*For each regression, we controlled for age, sex, CSF AD pathology, years of education, and APOE ε4 status.*

*AD: Alzheimer’s disease; APOE: Apolipoprotein E; CDR-SB: Clinical Dementia Rating scale sum of boxes score; CSF: Cerebrospinal fluid; NPI-Q: Neuropsychiatric Inventory Questionnaire*
